# Using population-scale medication data to evaluate the impact of the COVID-19 pandemic on the usage of analgesics by cancer patients.

**DOI:** 10.23889/ijpds.v7i3.2108

**Published:** 2022-08-25

**Authors:** Jun Han, Ashley Akbari, Fatemeh Torabi, Rowena Griffiths, Jane Lyons, Martin Rolles, Catherine Arnold, Dyfed Wyn Huws, Mark Lawler, Ronan Lyons

**Affiliations:** 1 Population Data Science, Health Data Research UK, Swansea University, Wales, UK; 2 DATA-CAN UK; 3 Swansea University Health Board, Swansea, Wales, UK; 4 DATA-CAN, The Health Data Research Hub for Cancer, UK; 5 Welsh Cancer Intelligence and Surveillance Unit, Public Health Wales, Cardiff, Wales, UK; 6 Queens University Belfast, Belfast, Northern Ireland, UK

## Objectives

Various analgesics are frequently prescribed to cancer patients for whom pain contributes to poor physical and emotional health and well-being. We examined changes in trends of analgesic prescribing in over 35,000 cancer patients diagnosed in the Welsh population before and during the COVID-19 pandemic in order to gain insight into the COVID-19 pandemic effects on cancer patients’ ability to receive analgesia and their potential ability to control their pain via medications.

## Approach

Within the Secure Anonymised Information Linkage (SAIL) Databank trusted research environment (TRE), patients diagnosed with incident primary breast, lung, colorectal or prostate cancers during 2017–2021 were obtained from Cancer Network Information System Cymru (CaNISC) dataset and patients’ prescription records were identified from Welsh Longitudinal General Practitioner (WLGP) dataset before being linked to their oncology e-record. We calculated opioid and non-opioid analgesic items prescribed per patient per year (PPPY) since cancer by clinical and demographic factors including cancer type, stage at diagnosis, diagnosis year, age at diagnosis, sex, comorbidities and patients’ socioeconomic status. These factors were included to model the effects of the COVID-19 pandemic on trends in analgesic prescribing for each cancer group.

## Results

We detected significant differences in the number of analgesic items prescribed PPPY in patients diagnosed before the COVID-19 pandemic (2017–2019) and those during the pandemic (2020–2021), with 1.3 more items PPPY prescribed for the latter group (p<0.001). Differences were accounted for largely by prescriptions for lung cancer patients, having 2.74 more items PPPY prescribed (p<0.001), the highest among the four cancer types evaluated. Patients diagnosed with a late-stage cancer had significantly more items prescribed than patients diagnosed at an early stage (p<0.001), with stage IV patients having 15.7 opioid items PPPY prescribed. For patients diagnosed at stage I, this rate PPPY was 6.7. Significant differences were also identified between patients from different socioeconomic backgrounds (p<0.001), with patients from the most deprived areas prescribed 11.3 items PPPY, 5.8 more than those from the least deprived areas.

## Conclusion

The significant impact of COVID-19 pandemic on pain medication prescribing for cancer patients could be partly related to the impact of COVID-19 lockdowns on presentation, waiting lists and diagnosis timings, and access to healthcare for prescriptions after diagnosis. Explanatory factors revealed by this study can help inform policymakers and provide guidance in improving pain relief for cancer services.

